# Current Situation of Transfusion-Transmitted Malaria in China

**DOI:** 10.1155/2021/3970370

**Published:** 2021-07-08

**Authors:** Hong Lin

**Affiliations:** Jiangsu Province Blood Centre, Nanjing 210042, China

## Abstract

Although China is moving toward the eradication of malaria and no indigenous malaria has been reported in most Chinese provinces for several years, recent evaluations have revealed that imported cases remain a major challenge to eliminating malaria, with the number of transfusion-transmitted malaria (TTM) cases increasing over time. Here, we review several TTM case reports published after the implementation of the National Malaria Elimination Program in 2010. A total of 12 TTM cases were reported in China between 2013 and 2018. All recipients and donors were diagnosed using rapid diagnosis test and peripheral blood smears. *Plasmodium* species in donors with low-density parasites were identified using PCR. Nine (75.0%) were identified as *Plasmodium falciparum*, two (16.7%) were identified as *Plasmodium vivax*, and one (8.3%) was identified as *Plasmodium ovale*. All were imported from malaria-endemic areas. New action plans designed to meet the challenges of TTM are necessary to ensure the elimination of malaria in China. Paying more attention to the frequency of TTM could help to enhance blood safety in China.

## 1. Introduction

Malaria is an important parasitic disease worldwide, which threatens the health and safety of various populations and whose infections slow both economic and social development. According to the WHO malaria report, there were 229 million malaria cases and more than 400,000 deaths globally in 2019 with the majority of these distributed in Sub-Saharan Africa and Southeast Asia [1]. Globally, the incidence of malaria has decreased rapidly. There are a growing number of countries who are moving towards malaria elimination. However, imported malaria cases pose a significant challenge to achieving these elimination goals [2]. Human malaria is caused by five species of the intraerythrocytic protozoan *Plasmodium*, including *P. falciparum*, *P. vivax*, *P. ovale*, *P. malariae*, and *P. knowlesi. Falciparum* malaria is the most prevalent and severe. *Plasmodium* species are always transmitted by the bite of *Anopheles* mosquitoes from an infected person to healthy subjects [3]. This is the most common mode of transmission in endemic areas. Other transmission routines include vertical transmission from infected mothers to their unborn babies (congenital malaria), blood transfusion, and organ or tissue transplantation, due to asymptomatic reservoirs of *Plasmodium* spp. presenting with low parasitaemia. These infections may result in transfusion-transmitted malaria (TTM) which may be fatal if not diagnosed timely and treated effectively [4]. A systematic review of TTM from 1971 to 2016 in the Americas showed that there were 422 cases of TTM described in 63 publications over this timeframe [5]. In another review, 100 case reports of TTM were retrieved from nonendemic areas with 54 cases reported on the American continent, 38 in Europe, 3 in the Mediterranean area, 1 in India, and 4 in Southeast Asia [6]. None of these TTM cases were from China likely because of the language barrier. During the long battle against malaria, numerous TTM cases have been reported in Chinese medical literature.

In the past, China suffered seriously from malaria epidemics, which makes it difficult to determine accurate statistics concerning total malaria cases, including TTM, till the foundation of the People's Republic of China in 1949. In addition, the lack of a complete information reporting system before the appearance of severe acute respiratory syndrome (SARS) in 2003 has also inhibited these evaluations [7]. Therefore, based on an incomplete statistical estimate, we can hypothesize that there were an estimated 6.97 million malaria patients nationwide (Taiwan not included), with an annual incidence rate of 12.29/1000 in 1954 [8]. It is worth noting that the majority of current TTM cases identify *P. vivax* as the causative agent. When the National Malaria Control Program was launched, the number of malaria cases declined dramatically. However, in the late 1980s and early 1990s, China experienced several blood donor-based malaria outbreaks in various parts of China, especially in those areas where natural transmission had been eliminated for several years [9]. Thousands of commercial plasmapheresis stations were established around the country, mostly in rural areas, and maintained by the companies producing blood-related products. However, the management of many illegal commercial plasmapheresis stations was chaotic, and cross-contamination from repeated needle use and a lack of sterile equipment resulted in an increase in the presence of several infectious agents, including HIV, malaria, and HCV, in the blood of the other donors [9, 10]. Some large scales of outbreaks of TTM from indigenous *Plasmodium* spp. were reported between 1993 and 1995 with the majority of cases in Hebei, Jiangsu, Henan, and Sichuan [11, 12]. The incidence of *P. falciparum* among paid plasmapheresis blood donors was significantly higher than that of residents and whole blood donors [12]. During the same period, there were some scattered reports of TTM caused by *P. vivax* and *P. ovale* in Guangdong, Guangxi, Yunnan, Heilongjiang, and other places [13]. Under the supervision of the Chinese government, commercial plasmapheresis stations were closed, and the Blood Donation Law of the People's Republic of China was implemented in 1998, resulting in improved control of TTM. The Chinese government then decided to embark on the national malaria elimination program (NMEP) in 2010 to support the government's goal of eliminating malaria by 2020. In the same year, the corresponding Action Plan for Chinese Malaria Elimination (2010–2020) (APCME) was officially implemented. By 2010, several decades of active prevention and control had already reduced the number of malaria cases to only 7855 nationwide, reducing the incidence rate to just 0.06 in one million [8]. The data suggests that the government's action plan is very effective as there have been no local malaria cases in most Chinese areas over the last several years (from 2014) [14], and no indigenous malaria cases were reported in China in 2017 for the first time; this means that all malaria cases were imported malaria cases [15].

The incidence of imported malaria is increasing and poses a serious threat to total malaria elimination in China. Due to global economic integration, people travel to malaria-endemic countries for trade, tourism, labour, and other purposes. The number of imported malaria cases has continued to rise on an almost daily basis in almost all provinces in China, with more than 2000 cases of imported malaria diagnosed every year for the past decade or more [16]. These imported *Plasmodium* spp. reservoirs present a unique challenge for blood banks, and infections of this kind can evolve into severe cases. To address these new challenges to blood safety in light of the malaria elimination goals, the main objective of this paper is to comprehensively review any TTM cases reported between 2011 and 2020 and characterize the epidemiology of TTM and provide some suggestions for reducing TTM and supporting the Chinese malaria elimination goals.

## 2. Methods

We examined all publicly available TTM case reports produced between January 2011 and March 2021 in the Chinese literature databases, including CNKI (China National Knowledge Infrastructure, http://www.cnki.net), Wanfang Data (http://www.wanfangdata.com), and the China Science and Technology Journal Database (CSTJ; http://www.cqvip.com). The keywords for the literature search included “transfusion transmitted malaria OR Blood transfusion AND malaria OR Blood transfusion AND plasmodium.” All pertinent case reports were downloaded and analysed in the context of the epidemiology of the disease in China. English papers were also identified using the PubMed database. The following data were extracted from each study: province, sex, and age of blood donor and recipient, occupation of blood donors, diagnostic method employed, blood donor screening method, blood component transfused, *Plasmodium* species, *Plasmodium* spp.'s original country, incubation period (i.e., the time elapsed between the infected transfusion and the onset of clinical symptoms in the recipient), and delayed diagnosis (i.e., time elapsed between the onset of clinical symptoms and the diagnosis of malaria).

## 3. Results

### 3.1. TTM Cases

A total of 21 papers reported cases associated with TTM in Chinese patients, only five of which seem to be duplicated. In one report, the recipient had not developed malaria after being transfused with 200 ml of blood contaminated with *P. falciparum* from a blood donor who had travelled to and from The Democratic Republic of the Congo (the Congo (King)) [17]. In total, there were 12 case reports for TTM published between 2014 and 2020 (Table 1) [18–28]. All 12 cases were written in Chinese, and only five abstracts (Cases 1–3, 6, and 8) were shown to be available in English on PubMed. With the exception of Case 12 reported briefly [28] and without clear details and Case 9 identified in a local CDC report which had not been published, the other 10 cases were reported with detailed descriptions of the disease development in the patients and characterisations of the infection *Plasmodium* spp.

### 3.2. Infected Malaria Patients

Of the 12 TTM cases, seven were female and five were male. The ages of the TTM cases ranged from 19 to 85 years, and they shared several common characteristics. First, none of the patients had a history of travel to a malaria-endemic country, and none had a history of infection. Second, all patients were transfused with red cell suspension prepared from infected blood donors. No corresponding plasma transfused patient was found to infect malaria. Third, the patients were not diagnosed in time with several patients experiencing a delay between fever onset and diagnosis, with the shortest period 3 days and the longest over a month. Fourth, the sources of the *Plasmodium* parasites were all imported from endemic countries, such as those in Africa and Southeast Asia. One older patient died (Case 6) due to his illness, and the others recovered successfully.

### 3.3. Asymptomatic Donors

The female-to-male ratio was 1 : 6 for blood donors. All were first-time donors. They were all young people, with a mean age of 27 years (range, 19–38 years), and all had a history of malaria or were living in an endemic area. The mean gap between their return to China and their donation was not more than 1 year (range, 3 days to more than 3 years). These donors included 8 employees dispatched abroad and 4 overseas students. A total of five of the donors (Cases 2, 8, 9, 10, and 12) had no malaria symptoms, and no parasite could be observed using microscopy (italics in Table 2) even when they were followed up. Others (58.3%) had no malaria symptoms when they donated, but they had typical malaria symptoms lasting three or more days following donation.

### 3.4. Laboratory Diagnosis

The diagnostic methods used for malaria screening and diagnosis are summarised in detail in Table 2. All malaria cases were investigated regularly, starting from malaria recipients to blood donors using a rapid diagnostic test (RDT). A total of 10 recipients were positive by RDT, and no RDT was performed in Cases 4 and 5. In addition, six donors were RDT negative, four were RDT positive, and two were not detected. The recipients in these cases were all positive for *Plasmodium* spp. in their red blood cells when evaluated using a microscope. When the donors were traced, eight had developed malaria, and parasites could be detected in their red blood cells; however, the other five donors (Cases 2, 8, 9, 10, and 12) had no symptoms and remained parasite free under the microscope. All cases, with the exception of Case 4, were confirmed to be malaria DNA positive by PCR. These PCRs included normal PCR, nested-PCR (nPCR), and real-time fluorescence quantitative PCR (RT-fqPCR). In addition, the donors in Cases 2 and 12, which had no detectable parasites under the microscope, produced positive results using more sensitive nested-Multi PCR. A total of 9 (75%) of the cases were shown to be *P. falciparum*, 2 (16.7%) were *P. vivax*, and 1 (8.3%) was *P. ovale* (Table 2).

## 4. Discussion

The elimination of malaria has been a top priority for the People's Republic of China since 1949 with the hope of reaching total eradication by 2020. This dedicated approach has seen China make tremendous progress in establishing effective control of the malaria epidemic over the last few decades. However, imported malaria cases pose a significant challenge to malaria elimination and pose a risk to blood safety. TTM has emerged as a sizeable challenge to malaria control in recent years, especially in provinces where foreign travel is more common (Table 1) and where there have been no indigenous cases of malaria for several years. Most of the donors identified in the TTM cases were adult males and had a labour-related travel history forcing them to spend time in other malaria-endemic countries. These donors were in a state of semi-immunity, and some of them carried an almost undetectable level of circulating parasites resulting in a lack of clear clinical symptoms of infection.

In theory, if the infected blood product is able to directly transmit the erythrocytic stage of the parasite to the recipient during TTM, the incubation period should be shorter than that in natural mosquito-transmitted malaria (MTM). In MTM, parasites experience a liver stage, where they mature over 5–16 days depending on the parasite species, and then infect red blood cells over 2-3 days leading to the development of erythrocytic schizonts, eventually destroying the erythrocyte [29]. In a report by Dover and Schultz [30], the incubation time was longer (16.0 d) in *P. falciparum* TTM than in its MTM infection (13.1 d). A global TTM review showed that these results were largely consistent throughout the world and that the mean incubation time for *P. falciparum* TTM was 25.7 days [6]. This might be explained by the low density of parasites in the asymptomatic donors, which require more time to mature [30]. In another review of TTM cases in the USA, the incubation time of *P. falciparum* TTM was 12.2 ± 4.1 days (range: 7–16 d) [31], which is shorter than that described for MTM. In our review, the incubation period was around 9 days for most TTM cases and increased to around 10.6 days (range: 3–23 d) in *P. falciparum* TTM. This may be because most of the donors presented with more robust subclinical infections which allowed the parasites to be observed using microscopy and meant that the parasite inoculums were sufficient to establish clinical symptoms in a shorter period of time.

As reviewed, 30.6% malaria cases were delayed in their diagnoses, often exceeding the more common 3-day timeframe in clinical interventions [6]. Unfortunately, these delays may have contributed to the death of some patients due to delayed treatment. In our review, it took approximately 15 days (range, 3–39 d) from fever to diagnosis. Although routine blood tests for some infectious agents are mandatory in hospitalised patients in China, malaria screening is not routinely performed. Patients who need blood transfusions are always treated as critical and severe, and some of their initial symptoms may overlap with those of malaria. This means that the addition of several atypical malaria symptoms made it difficult to diagnose and resulted in a delay. In China, the significant decline in malaria cases has meant that professionals who engage in malaria prevention and treatment are not always as familiar with its clinical presentation as hoped. Medical staff, especially young physicians, lack awareness, skills, and experience in malaria management and diagnosis. In some hospitals, there are no departments specialising in malaria prevention and treatment, and patients need to visit other hospitals. This often leads to misdiagnosis or missed diagnosis reducing the efficacy of the clinical interventions resulting in increased disease burden and even death. Therefore, given the rise in imported malaria, it is critical that the support training should strengthen the identification, diagnosis, and treatment of malaria by younger, less experienced physicians [16].

Blood banks usually evaluate the donor candidates' health before donation, including screening for current infections, and a detailed history of previous disease and travel. In China, donor candidates should meet the Blood Donor Physical Examination Requirements (GB18467-2011) which include weight, blood pressure, and haemoglobin evaluation using the copper sulphate method, alanine aminotransferase (ALT) activity, and HBV surface antigen testing by colloidal gold strip. However, before these evaluations, donors are required to complete the Health Check Questionnaire, which itemizes disease and travel history, and sign informed consent. For malaria, it rules that those who have had a diagnosis of malaria or lived in an endemic country for 12 months are deferred to donate for 3 years after becoming asymptomatic. Therefore, the donors in this review should have been prevented from donating for between one and three years following their return to being asymptomatic. In practice, these guidelines were not properly applied. These donors intentionally or unintentionally concealed or forgot their malaria history. Some foreign donors may not fully understand the questionnaire due to the language barrier. In most areas of China, indigenous malaria has been eliminated for more than 20 years. The public's perception of risk for malaria is very low, and many younger donors have not even heard of this disease [32]. Most of them lack some degree of awareness, knowledge, and precautionary measures for dealing with malaria. Many of the interviewing staff also neglect potential malaria infections and are unaware of the danger of malaria infection to recipients. It was estimated that approximately 70% of the cases of TTM occurred due to a failure in donor deferral during the screening questionnaire interview [33]. Therefore, identifying these donors is critical to ensuring the safety of blood products in China and places a significant burden on clinical and interviewing staff, making their recruitment a challenge.

Effective screening of the donations can also significantly decrease the risk of TTM. Microscopy, detection of plasmodia antibodies or antigens by immunology assay, or plasmodial DNA by PCR can all be applied to screen donations reducing the risk of contamination. In a systematic review and meta-analysis [3], the median prevalence of malaria parasitaemia among 984975 asymptomatic healthy blood donors was 10.54%, 5.36%, and 0.38% as determined by microscopy, PCR, and rapid diagnostic tests (RDTs), respectively. The detection of plasmodial DNA by PCR has been shown to provide relatively high levels of sensitivity and can detect 0.002 parasites/*µ*L [34], but not every laboratory can achieve this degree of precision. We found that normal PCR in some laboratories could not detect plasmodial DNA in low-density parasite donors (Table 2). Despite the fact that nucleic acid testing (NAT) is employed by most blood banks around the world, including China, for the detection of RNA or DNA from a host of diseases including HIV, HCV, and HBV, NAT is a useful tool for facilitating malaria control and elimination efforts [35]. That said, there has been little research on its practical application for malaria screening in blood banks. A prototype HIV/HCV/HBV/malaria NAT assay was developed and validated in Brazil and has been shown to be a promising alternative for screening for malaria in blood banks in both endemic and nonendemic regions [36]. Despite this, the cost and infrastructure demands of this method still prevent its effective application in routine screening. However, it may be feasible to use PCR as a selective screening in high-risk donors in some developing countries. Improving molecular diagnostic tools and their application in combination with control strategies would lead to making elimination of malaria from global landscape to a practical reality in low transmission settings [37]. In our blood centre, in-house nested-PCR [38] is used to screen the high-risk donors who are from malaria-endemic areas.

Jiangsu, located in eastern China, was once an endemic home for *P. vivax*. Indigenous vivax malaria has been effectively controlled through the comprehensive implementation of effective interventions [39]. No indigenous malaria cases have been reported since 2012 [38, 40]. In 2019, Jiangsu Province passed the final assessment of the national elimination of malaria program and still had the highest number of imported malaria cases in China [41]. Given the fact that the source of infection (imported cases from abroad) cannot be completely eliminated and that the main malaria vector, the *Anopheles* mosquitoes, has not been eradicated, Jiangsu Province continues to implement stringent criteria for malaria infection reporting. It is the first province to implement the 1-3-7 surveillance and response strategy (reporting of malaria cases within one day, their confirmation and investigation within three days, and the appropriate public health response to prevent further transmission within seven days) [42], which traces the source of every malaria case, including TTM cases [43]. This has been implemented nationwide and is recommended by the WHO as an example for malaria surveillance and elimination [44]. This is the reason why more TTM cases are identified in Jiangsu Province than in the other provinces in China. Some mitigation strategies designed to reduce TTM were also recommended to the Jiangsu blood banks, and a guideline for malaria prevention was set in place in April 2017. The first barrier to the effective implementation of these processes remains the blood donor questionnaire. The Jiangsu provincial blood centres went on to produce a series of malaria education courses designed to train the interview staff at the blood bank to recognise potential risks and defer donors more effectively. No donation is collected from any overseas students or workers within one year of their arrival in or return to China. These guidelines advocate the use of in-house nested-PCR [38] and real-time fluorescence quantitative PCR [45] to screen returning personnel or foreigners from malaria-endemic areas of Africa and Southeast Asia. These guidelines also recommend that only *Plasmodium* species negative blood components be used in the clinic.

## 5. Conclusion

China launched its malaria elimination program in July 2010, with a plan to achieve total eradication by 2020. Although many malaria research papers have been published in China, TTM has been largely neglected. The challenges faced in TTM mirror the considerations in reducing national malaria risk and improving general blood safety in China. To mitigate the risk of TTM as much as possible, knowledge gaps in TTM need to be highlighted and addressed in the pursuit of malaria elimination, and public health authorities should consider how to deploy the best blood safety surveillance strategies. Currently, there are no mandatory malarial agent screening methods available to Chinese blood banks. The first and only defence against TTM is strengthening our blood donor management and using questionnaire-based risk assessment. In areas where the number of imported malaria cases is increasing, it is advised that high-risk blood should be selectively screened by in-house PCR.

## Figures and Tables

**Table 1:** Demographic data for donors and recipients in the 12 TTM cases.

Case	Year	Province	Donor				Recipient					References
			Gender	Age	Occupation	From coming/back to China to donation (days)	Gender	Age	Incubation (days)^*∗*^	Delayed diagnosis (days)^*∗∗*^	Outcome	
1	2013	Zhejiang	F	21	Worker	101	F	45	23	12	Recovery	[18]
2	2013	Shanghai	M	25	Overseas student	>3 years	M	61	25	41	Recovery	[19]
3	2013	Jiangsu	M	33	Engineer	160	M	28	3	11	Recovery	[20]
4	2015	Guangdong	M	37	Worker	>1.5 years	F	37	13	6	Recovery	[21]
5	2015	Guangxi	M	29	Engineer	292	M	81	16	6	Recovery	[22]
6	2016	Jiangsu	F	19	Overseas student	3	M	64	17	18	Death	[23]
7	2017	Jiangsu	M	21	Overseas student	197	F	43	13	10	Recovery	[24]
8	2017	Guangdong	M	20	Overseas student	>1 year	M	19	2	21	Recovery	[25]
9	2017	Jiangsu	M	38	Worker	31	F	73	10	23	Recovery	^*∗∗∗*^
10	2018	Hunan	M	^*∗∗∗∗*^	Worker	107	F	42	8	3	Recovery	[26]
11	2018	Shandong	M	^*∗∗∗∗*^	Trader	53	F	33	6	18	Recovery	[27]
12	2018	Shanxi	M	^*∗∗∗∗*^	Overseas student	>1 year	F	62	23	^*∗∗∗∗*^	Recovery	[28]

^*∗*^Incubation period refers to “days from transfusion to fever.” ^*∗∗*^Delayed diagnosis time means “days from fever to confirmation.” ^*∗∗∗*^Unpublished and according to the data from the local CDC. ^*∗∗∗∗*^Unknown.

**Table 2:** Plasmodium detection and species identification in the 12 Chinese cases of TTM.

Case no.		Parasite detection			Plasmodium	
		RDT	Microscopy	PCR	Species	Source
1	D	NA	＋	nPCR/＋	*P. falciparum*	Equatorial Guinea
	R	＋	＋	nPCR/＋		

2	*D*	−	−	*nMulti-PCR/＋*	*P. vivax*	*Cote d'Ivoire*
	*R*	＋	＋	*nPCR/＋*		

3	D	−	＋	Rt-fqPCR/＋	*P. falciparum*	Equatorial Guinea
	R	＋	＋	Rt-fqPCR/＋		

4	D	＋	＋	—	*P. falciparum*	Cameroon
	R	NA	＋	—		

5	D	NA	＋	PCR/＋	*P. ovale*	Equatorial Guinea
	R	NA	＋	PCR/＋		

6	D	−	＋	Rt-fPCR/＋	*P. falciparum*	Indonesia
	R	＋	＋	Rt-fPCR/＋		

7	D	−	＋	Rt-fPCR/＋	*P. falciparum*	Nigeria
	R	＋	＋	Rt-fPCR/＋		

8	*D*	＋	−	*nPCR/＋*	*P. falciparum*	*Sudan*
	*R*	＋	＋	*nPCR/＋*		

9	*D*	＋	−	*Rt-fPCR/＋*	*P. falciparum*	*Equatorial Guinea*
	*R*	＋	＋	*Rt-fPCR/＋*		

10	*D*	＋	−	*nPCR/＋*	*P. falciparum*	*Cameroon*
	*R*	＋	＋	*nPCR/＋*		

11	D	＋	＋	PCR/＋	*P. falciparum*	Ghana
	R	＋	＋	PCR/＋		

12	*D*	−	−	*nMulti-PCR/＋*	*P. vivax*	—
	*R*	＋	＋	*nPCR/＋*		

D, donor; R, recipient; NA, not applicable; —, unknown; nPCR, nested PCR; nMulti-PCR, nested-Multi PCR; Rt-fqPCR, real-time fluorescence quantitative PCR.

## Data Availability

The data used and analysed during the current study are included within the manuscript.
